# Two *BRM* promoter insertion polymorphisms increase the risk of early-stage upper aerodigestive tract cancers

**DOI:** 10.1002/cam4.201

**Published:** 2014-02-12

**Authors:** Kit Man Wong, Xiaoping Qiu, Dangxiao Cheng, Abul Kalam Azad, Steven Habbous, Prakruthi Palepu, Maryam Mirshams, Devalben Patel, Zhuo Chen, Heidi Roberts, Jennifer Knox, Stephanie Marquez, Rebecca Wong, Gail Darling, John Waldron, David Goldstein, Natasha Leighl, Frances A Shepherd, Ming Tsao, Sandy Der, David Reisman, Geoffrey Liu

**Affiliations:** 1 Department of Medical Oncology, Princess Margaret Cancer Center, University of TorontoToronto, Ontario, Canada; 2 Institute of Virology, Medical School of Wuhan UniversityWuhan, Hubei, China; 3 Ontario Cancer Institute, Princess Margaret Cancer Center-University Health NetworkToronto, Ontario, Canada; 4 Department of Radiology, Princess Margaret Cancer Center, University of TorontoToronto, Ontario, Canada; 5 Division of Hematology/Oncology, Department of Medicine, University of FloridaGainesville, Florida; 6 Department of Radiation Oncology, Princess Margaret Cancer Center, University of TorontoToronto, Ontario, Canada; 7 Department of Thoracic Surgery, Princess Margaret Cancer Center, University of TorontoToronto, Ontario, Canada; 8 Department of Otolaryngology-Head and Neck Surgery, Princess Margaret Cancer Center, University of TorontoToronto, Ontario, Canada; 9 Department of Laboratory Medicine, Princess Margaret Cancer Center, University of TorontoToronto, Ontario, Canada; 10 Dalla Lana School of Public Health, University of TorontoToronto, Ontario, Canada

**Keywords:** *BRM*, cancer risk, case–control study, esophageal cancer, genetic polymorphisms, head and neck cancer, lung cancer, upper aerodigestive tract cancers

## Abstract

Brahma (BRM) has a key function in chromatin remodeling. Two germline *BRM* promoter insertion–deletion polymorphisms, *BRM*-741 and *BRM*-1321, have been previously associated with an increased risk of lung cancer in smokers and head and neck cancer. To further evaluate their role in cancer susceptibility particularly in early disease, we conducted a preplanned case–control study to investigate the association between the *BRM* promoter variants and stage I/II upper aerodigestive tract (UADT) cancers (i.e., lung, esophageal, head and neck), a group of early-stage malignancies in which molecular and genetic etiologic factors are poorly understood. The effects of various clinical factors on this association were also studied. We analyzed 562 cases of early-stage UADT cancers and 993 matched healthy controls. The double homozygous *BRM* promoter variants were associated with a significantly increased risk of early stage *UADT* cancers (adjusted odds ratio [aOR], 2.46; 95% confidence interval [CI], 1.7–3.8). This association was observed in lung (aOR, 2.61; 95% CI, 1.5–4.9) and head and neck (aOR, 2.75; 95% CI, 1.4–5.6) cancers, but not significantly in esophageal cancer (aOR, 1.66; 95% CI, 0.7–5.8). There was a nonsignificant trend for increased risk in the heterozygotes or single homozygotes. The relationship between the *BRM* polymorphisms and early-stage UADT cancers was independent of age, sex, smoking status, histology, and clinical stage. These findings suggest that the *BRM* promoter double insertion homozygotes may be associated with an increased risk of early-stage UADT cancers independent of smoking status and histology, which must be further validated in other populations.

## Introduction

Epigenetic regulation of gene expression may occur by histone deacetylation and methylation, cytosine methylation, and chromatin remodeling 1,2. Altered epigenetic regulation affects normal gene transcription and is potentially tumorigenic. The SWI/SNF (SWItch/sucrose nonfermentable) complex is an ATP-dependent chromatin remodeling complex that has been shown to modulate gene expression and mediate many important cellular processes, including cell cycle, growth and differentiation, DNA repair, and cell adhesion 3–12. This multimeric complex promotes gene expression by shifting the positions of histones in the chromatin to facilitate DNA access by transcription factors 13. SWI/SNF is involved in regulating several key tumor suppressor genes, such as *RB*, *p53*, and *BRCA* 5,14, and impaired function of SWI/SNF is associated with lung cancer development 15.

Brahma (BRM) is one of two catalytic ATPase subunits essential for the function of the SWI/SNF complex, and there is mounting evidence that *BRM* is a tumor suppressor gene 3,16. Loss of heterozygosity of the *BRM* locus (9p23-24) occurs in a variety of malignancies 17–21. BRM protein expression is absent in 40% of lung cancer cell lines and in 18% of primary lung tumors irrespective of histology 22,23. Moreover, BRM is repressed in 10–20% of other cancers, including breast, colon, esophageal, gastric, head and neck, ovarian, prostate, and bladder cancers 23–26. Further support for its tumor suppressor effects comes from in vitro evidence of growth inhibition of BRM-deficient cell lines by the reexpression of BRM 27,28. The loss of BRM is also associated with poorer prognosis in nonsmall cell lung cancer, supporting its role in lung cancer pathogenesis and progression 22,29.

BRM expression has been shown to be epigenetically regulated 23,30. The sequencing of the *BRM* promoter in BRM-deficient lung cancer cell lines and primary lung tumors identified two novel germline insertion variants, *BRM*-741 (rs34480940; 7 bp indel [insertion–deletion] polymorphism) and *BRM*-1321 (rs3832613 or rs59259177; 6 bp indel polymorphism), that are postulated to recruit MEF2 and histone deacetylases 15. The presence of both homozygous polymorphisms strongly correlated with loss of BRM expression in primary lung tumors (*P* = 0.015), as well as adjacent normal lung tissue (*P* = 0.002). Furthermore, in a case–control analysis of 1119 smokers, double homozygosity for the *BRM* promoter variants was most strongly associated with the risk of lung cancer independent of histology (adjusted odds ratio [aOR], 2.19; 95% confidence interval [CI], 1.40–3.43; *P* = 0.0006) 15. Given that only a subset of smokers develops lung cancer, these results raised the possibility that *BRM*-741 and *BRM*-1321 increase the risk of malignancy in predisposed individuals with prior carcinogenic exposure. In addition, another case–control study from our group demonstrated that homozygosity for the *BRM* promoter polymorphisms increased the risk of head and neck squamous cell carcinoma, particularly for the double homozygotes (aOR, 2.23; 95% CI, 1.5–3.4; *P* < 0.001) 31. In the aforementioned studies, patients of all stages were included, with subgroup analyses suggesting that the BRM-risk association may be stronger in more advanced disease.

The three upper aerodigestive tract (UADT) cancers (i.e., lung, esophageal, head and neck) are frequently diagnosed at advanced stage with poor prognosis 32. Their molecular and genetic etiologic factors are poorly understood. In fact, there is a need to better understand the factors predisposing to early-stage UADT cancers in order to improve screening strategies. Given the earlier associations between the *BRM* promoter variants and lung cancer among smokers and head and neck cancer across all stages 15,31, we sought to determine whether *BRM*-741 and *BRM*-1321 are similarly correlated with esophageal cancer, to characterize the BRM-risk association specifically in early-stage UADT malignancies, as well as to assess whether the increased risk of malignancy is restricted to ever-smokers. Unlike the previous studies that included any clinical stage, this analysis specifically focused on patients with stage I/II tumors, as the aim was on investigating the genetic risk of early-stage cancer and identifying potential risk biomarkers that may be useful in early detection. To this end, we conducted a preplanned case–control study to investigate the correlation between the *BRM* promoter variants and early-stage UADT cancers, as well as the factors that influence this association, including smoking status and histology. All of our analyses involved cases and controls that have not been previously evaluated in our prior studies, and thus also serve as confirmatory analyses.

## Materials and Methods

### Patients and data/sample collection

A total of 562 cancer patients with histologically proven stage I/II UADT cancers treated at Princess Margaret Cancer Center (PMCC, Toronto, Ontario, Canada, 2001–2006) were part of a molecular epidemiologic study of cancer risk and prognostic factors, and were included in the analysis. These cases consisted of 268 lung, 110 esophageal, and 184 head and neck cancers. Eligibility criteria included age 18 years or older, ability to communicate in English, self-reported Caucasian ancestry, and lack of cognitive deficits to ensure that participants had an understanding of the study. Non-Caucasians represented a small subset of the overall population and were excluded to reduce bias from population stratification. Lung cancer and head and neck cancer cases and controls formerly included in Liu et al. 15 and Wang et al. 31, respectively, were excluded from the current analysis. We restricted all UADT cases to adenocarcinoma (i.e., lung and esophageal) or squamous cell carcinoma (i.e., lung, esophageal, and head and neck); large cell carcinoma of the lung that was not classified as large cell neuroendocrine tumor was also included.

A total of 993 healthy controls were matched to the 562 cases by frequency distribution according to age, sex, and smoking status. For each case, we identified two matching controls of the same sex and smoking status, with their mean age equal to that of the case of interest. Screening controls who were smokers (*n* = 650) were chosen from the Lusi Wong Early Detection of Lung Cancer Screening Program (PMCC), which enrolled over 3900 patients. These individuals from the same catchment area as the cases responded to notices posted in Toronto hospitals and an unsolicited article in the largest local newspaper to participate in a screening program. On the other hand, nonsmoker screening controls (*n* = 343) were healthy friends of the cancer patients who responded to requests by volunteer recruiters to serve as controls for the study and lived in the catchment area of the cases. Participant criteria for the healthy controls in the cancer screening program included age 18 years or older, ability to speak English, and being genetically unrelated to known cases. Spouses of cancer patients were specifically excluded as controls for the current analysis. The epidemiologic study and screening research program described above were approved by the research ethics board at University Health Network, and all participants provided consent.

The Harvard Oncologic Molecular Epidemiologic (HOME) Survey, a standardized epidemiologic questionnaire of social habits and family history, was administered to all participants 33. Whole blood was collected from all participants at the time of enrollment and stored at −70°C.

### Genomic DNA extraction and sequencing

Genomic DNA was extracted from whole blood-derived lymphocytes of the 562 cases and 993 controls according to previously described protocols 15. Genotyping of the *BRM*-741 and *BRM*-1321 promoter insertion polymorphisms was conducted on extracted DNA by qPCR using TaqMan® probes (Life Technologies Inc., Burlington, Canada). The primers and PCR protocol used have been described previously 15.

### Statistical analysis

Baseline characteristics were tabulated for the cases and matched controls, and compared using chi-square and *t*-tests. All primary and subgroup analyses were preplanned. The risk of UADT cancers was analyzed by multivariate logistic regression using SAS version 9.3 to generate aORs, which were adjusted for age, sex, smoking status, pack-year history, and family history of UADT cancers. Subgroup analyses were performed with respect to age, sex, smoking status, family history of UADT cancers, disease site, histology, and clinical stage.

## Results

### Characteristics of the case and control populations

The 562 cases of early-stage UADT cancers included: 268 (48%) lung, 110 (20%) esophageal, and 184 (33%) head and neck cancers, which consisted mostly of oral (*n* = 93) and laryngeal (*n* = 72) cancers. Among these, 41% were adenocarcinomas and 55% were squamous cell carcinomas. The majority of patients had stage I disease (77%). The cases and controls were matched for age (mean 62 years), sex (63% male), and smoking status (23% current smokers, 43% ex-smokers, 34% never-smokers). The case and control populations had mean smoking histories of 44 and 29 pack-years, respectively. The characteristics of the cases and controls are shown in Table 1.

**Table 1:** Baseline characteristics of the cases and their matched controls.

Characteristic	Cases (*n* = 562)	Controls (*n* = 993)	*P*-value
Age, mean (range)	62 (18–92)	62 (30–87)	0.71
Sex, *n* (%)			
Male	352 (63)	624 (63)	0.97
Female	210 (37)	369 (37)	
Smoking status, *n* (%)			
Current smokers	129 (23)	226 (23)	0.65
Ex-smokers	240 (43)	424 (43)	
Never-smokers	193 (34)	343 (35)^1^	
Pack-year history, mean (range)	44 (0.1–218)	29 (2–190)	<0.0005
Family history of UADT cancers, *n* (%)			
Yes	23 (4)	39 (4)	0.60
No	539 (96)	954 (96)	
Cancer type, *n* (%)			
Lung	268 (48)		
Esophageal	110 (20)		
Head and neck	184 (33)^1^		
Histology, *n* (%)			
Adenocarcinoma	233 (41)		
Squamous cell carcinoma	309 (55)		
Large cell carcinoma	20 (4)		
Stage, *n* (%)			
I	435 (77)		
II	127 (23)		
ECOG performance status, *n* (%)			
0–1	469 (83)		
2 or greater	93 (17)		

UADT, upper aerodigestive tract.

1 Percentages do not add up to 100% due to rounding.

### The association between *BRM*-741 and *BRM*-1321 promoter polymorphisms and early-stage UADT cancers

The frequencies of the *BRM* promoter polymorphisms were determined in the cases and controls, and their association with early-stage UADT cancers was evaluated relative to the wild-type (Table 2). Homozygosity for *BRM*-741, *BRM*-1321, or both was observed in 32% and 28% of cases and controls, respectively. The risk of early-stage UADT cancers was significantly increased by more than twofold in patients with the double homozygous variants (aOR, 2.46; 95% CI, 1.7–3.8; *P* < 0.0001). In contrast, the heterozygotes and single homozygotes had a nonsignificant trend for increased risk, with aORs intermediate between those of the wild-type and double homozygote subgroups. When combined together, the heterozygotes and single homozygotes were found to have an increased overall risk of early-stage UADT cancers compared to wild-type (aOR, 1.39; 95% CI, 1.1–1.7; *P* = 0.03).

**Table 2:** Association between *BRM* promoter polymorphism and UADT cancers.

*BRM* polymorphism	Cases, *n* (%)	Controls, *n* (%)	Adjusted OR (95% CI)^1^; *P*-value
All cancers	*n* = 562	*n* = 993	
Wild type (reference)	87 (15)	205 (21)	1
Heterozygote (for either variant)	296 (53)	512 (52)	1.38 (1.0–1.8)
*BRM*-741 homozygote only	58 (10)	97 (10)	1.45 (0.9–2.2)
*BRM*-1321 homozygote only	66 (12)	114 (11)	1.39 (0.9–2.1)
*BRM*-741 and-1321 homozygotes	55 (10)	65 (7)^2^	2.46 (1.7–3.8)
Lung cancer	*n* = 261	*n* = 436	
Wild type (reference)	39 (15)	91 (21)	1
Heterozygote (for either variant)	137 (52)	223 (51)	1.45 (0.9–2.4)
*BRM*-741 homozygote only	28 (11)	45 (10)	1.48 (0.9–2.9)
*BRM*-1321 homozygote only	30 (11)	48 (11)	1.47 (0.8–2.7)
*BRM*-741 and-1321 homozygotes	27 (10)^2^	29 (7)	2.61 (1.5–4.9)
Esophageal cancer	*n* = 113	*n* = 155	
Wild type (reference)	20 (18)	30 (19)	1
Heterozygote (for either variant)	59 (52)	83 (54)	1.07 (0.5–2.2)
*BRM*-741 homozygote only	10 (9)	13 (8)	1.15 (0.4–3.6)
*BRM*-1321 homozygote only	14 (12)	18 (12)	1.18 (0.4–3.3)
*BRM*-741 and-1321 homozygotes	10 (9)	11 (7)	1.66 (0.7–5.8)
Head and neck cancer	*n* = 188	*n* = 402	
Wild type (reference)	28 (15)	84 (21)	1
Heterozygote (for either variant)	100 (53)	206 (51)	1.46 (1.0–2.4)
*BRM*-741 homozygote only	20 (11)	39 (10)	1.55 (0.7–3.2)
*BRM*-1321 homozygote only	22 (12)	48 (12)	1.42 (0.7–3.1)
*BRM*-741 and-1321 homozygotes	18 (10)^2^	25 (6)	2.75 (1.4–5.6)

*BRM*, Brahma; OR, odds ratio; CI, confidence interval; UADT, upper aerodigestive tract.

1 The OR was adjusted for: age, sex, smoking status, pack-years, and family history of UADT cancers.

2 Percentages do not add up to 100% due to rounding.

Separate analyses of the three UADT cancers showed that double homozygosity for the *BRM* variants was significantly correlated with lung (aOR, 2.61; 95% CI, 1.5–4.9; *P* = 0.006) and head and neck cancers (aOR, 2.75; 95% CI, 1.4–5.6; *P* = 0.004). On the other hand, there was a nonsignificant trend toward association between esophageal cancer and the double homozygotes (aOR, 1.66; 95% CI, 0.7–5.8; *P* = 0.31).

### The impact of clinical factors on the association between the *BRM* promoter variants and early-stage UADT cancers

**Figure 1:**
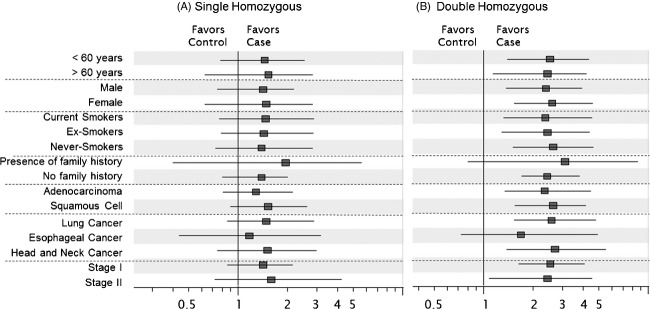
Impact of clinical factors on the association between the single homozygous (A) or the double homozygous (B) *BRM* promoter variants and early-stage UADT cancers. The ORs were adjusted for age, sex, smoking status, pack-years, and family history of UADT cancers. *BRM*, Brahma; OR, odds ratio; UADT, upper aerodigestive tract.

The effects of several clinical factors on the association between the *BRM* promoter polymorphisms and stage I/II UADT cancers were determined (Fig. 1 and Table S1). The magnitude of risk with the double homozygous *BRM* variants was not influenced by age, sex or smoking status. Moreover, the likelihood of cancer was similar for all histologies and clinical stages.

Previous studies of the *BRM* polymorphisms in cancer were limited to smokers. Therefore, the relationship between *BRM* genotype and UADT cancers was examined separately in ever-smokers and never-smokers (Table 3). The increased risk of malignancy in patients with *BRM*-741/-1321 double homozygosity was similar in ever-smokers (aOR, 2.38; 95% CI, 1.3–4.4; *P* = 0.02) and never-smokers (aOR, 2.62; 95% CI, 1.2–5.0; *P* = 0.04) (interaction *P* = 0.32). Moreover, the magnitude of cancer risk stratified by smoking status was similar in separate analyses of lung, esophageal, and head and neck cancers.

**Table 3:** The frequency of *BRM* polymorphisms in smokers and nonsmokers with upper aerodigestive tract cancers.

	Adjusted OR (95% CI)^1^ cases vs. controls		
*BRM* polymorphism	Ever-smokers	Nonsmokers	Interaction *P*-value
All cancers			
Wild type (reference)	1	1	0.32
Heterozygote (for either variant)	1.36 (0.8–2.2)	1.39 (0.7–2.6)	
*BRM*-741 homozygote only	1.26 (0.6–3.2)	1.43 (0.9–3.9)	
*BRM*-1321 homozygote only	1.25 (0.6–3.2)	1.35 (0.5–4.0)	
*BRM*-741 and-1321 homozygotes	2.38 (1.3–4.4)	2.62 (1.2–5.0)	
Lung cancer			
Wild type (reference)	1	1	0.55
Heterozygote (for either variant)	1.33 (0.5–3.7)	1.36 (0.6–4.7)	
*BRM*-741 homozygote only	1.38 (0.5–4.5)	1.42 (0.4–5.0)	
*BRM*-1321 homozygote only	1.66 (0.9–4.7)	1.25 (0.4–5.2)	
*BRM*-741 and-1321 homozygotes	2.40 (1.2–4.4)	2.49 (1.0–5.0)	
Esophageal cancer			
Wild type (reference)	1	1	0.88
Heterozygote (for either variant)	1.05 (0.4–3.6)	1.09 (0.3–4.1)	
*BRM*-741 homozygote only	1.15 (0.4–4.2)	1.13 (0.3–4.8)	
*BRM*-1321 homozygote only	1.22 (0.3–3.9)	1.00 (0.3–5.1)	
*BRM*-741 and-1321 homozygotes	1.81 (0.6–3.0)	1.52 (0.6–4.3)	
Head and neck cancer			
Wild type (reference)	1	1	0.42
Heterozygote (for either variant)	1.41 (0.7–4.2)	1.55 (0.8–5.2)	
*BRM*-741 homozygote only	1.51 (0.6–4.8)	1.58 (0.5–6.0)	
*BRM*-1321 homozygote only	1.56 (0.8–5.6)	1.47 (0.6–6.3)	
*BRM*-741 and-1321 homozygotes	2.53 (1.4–4.5)	3.15 (1.4–6.4)	

*BRM*, Brahma; OR, odds ratio; CI, confidence interval; UADT, upper aerodigestive tract.

1 The OR was adjusted for: age, sex, smoking status, pack-years, and family history of UADT cancers.

## Discussion

This case–control study found that double homozygosity for the *BRM* germline promoter insertion polymorphisms, *BRM*-741 and *BRM*-1321, was significantly associated with an increased risk of early-stage UADT cancers by more than twofold. This significant association was observed primarily in early-stage lung and head and neck cancers, while the magnitude and significance of the risk of esophageal cancer were lower. Furthermore, subgroup analyses showed that the increased risk of malignancy was independent of age, sex, smoking history, histology, and clinical stage.

Liu et al. 15 previously showed that the double homozygous *BRM* variants increased the risk of stages I–IV lung cancer among active and ex-smokers (aOR, 2.19; 95% CI, 1.4–3.4; *P* = 0.0006). This was validated in this study of early-stage lung cancer patients, which found a similar association between the double homozygotes and lung cancer risk (aOR, 2.61; 95% CI, 1.5–4.9; *P* = 0.006). In addition, this study expands our understanding of the etiologic relevance of the *BRM* promoter polymorphisms. First, the higher lung cancer risk of the *BRM* variants was observed in lifetime never-smokers, which suggests that these genetic polymorphisms confer risk independent of smoking status. The association was similar for lung adenocarcinomas and squamous cell carcinomas, despite the potentially different biological pathways in these histological subtypes 34. Moreover, a significant association between the double homozygotes and early-stage head and neck cancer was demonstrated, confirming the results of Wang et al. 31 in the early-stage subset. Thus, *BRM*-741 and *BRM*-1321 may be germline genetic variants relevant in both ever-and never-smokers, as well as across different cancers (lung, head and neck) and histological subtypes (adenocarcinoma, squamous cell carcinoma). While there are somatic genetic changes that are more prevalent in never-smoking lung cancer patients (e.g., *EGFR* mutations, *ALK* translocations 35), the *BRM* polymorphisms are potential germline biomarkers that may identify a subset of never-smokers with a twofold greater risk of lung cancer. However, further study of the role of *BRM* and its promoter polymorphisms in tumorigenesis, as well as validation of these genetic variants as biomarkers of cancer risk will be necessary in order to establish their clinical utility.

In addition, the association between the *BRM* promoter variants and UADT cancers observed in this study has potential therapeutic implications. While the double homozygous variants lead to the epigenetic loss of BRM expression in cancer cell lines and primary lung tumors, Gramling et al. demonstrated the pharmacologic recovery of BRM expression and function across BRM-deficient cell lines using two agents identified from a high-throughput drug screen 15,36. Although further study will be required to clarify the role of epigenetic BRM silencing as an oncogenic driver in the pathogenesis of UADT cancers, the current data raise the possibility of reversing this epigenetic dysregulation as a novel therapeutic and/or preventive approach in these malignancies.

We observed that the double homozygotes had a significantly greater risk of early-stage UADT cancers compared to the heterozygotes or single homozygotes. Interestingly, although the association was not significant, the aORs of the heterozygotes and single homozygotes were similar and intermediate between those of the wild-type and double homozygotes, suggesting the possibility of a gene-dose effect. It may be that the repression of BRM only occurs in the presence of both homozygous insertion alleles. 9q23-24 is an area highly affected by loss of heterozygosity in many tumors, and selective loss of the wild-type deletion alleles during carcinogenesis alongside linkage disequilibrium of the two polymorphisms may be driving the trend toward cancer association in individuals carrying the germline heterozygotes in one or both polymorphisms, as seen in the current and prior studies 15. Future molecular studies will be needed to evaluate the consequences of these promoter variant genotypes on BRM expression and their mechanisms in promoting cancer susceptibility.

This study has several limitations. First, the small number of esophageal cancer patients was underpowered to detect a smaller association of less than twofold with the double homozygous *BRM* variants. The study population consisted of only Caucasians and was derived from a single institution, which may affect the generalizability of the results. Our analysis also excluded small cell and large cell neuroendocrine lung cancers. Moreover, the control group was not population-based, as it was selected from a lung cancer screening program (smokers) and unrelated friends of other cancer patients (nonsmokers). Therefore, our findings need to be validated in future studies and in other patient populations.

In summary, we have shown that the double homozygous *BRM* germline variants are associated with an increased risk of early-stage UADT cancers. This increased cancer risk is not affected by prior smoking history, histology, and disease site, suggesting that these promoter polymorphisms may independently contribute to cancer susceptibility. Further studies are needed to understand the biology of the *BRM* promoter variants in carcinogenesis and to validate their clinical utility as potential biomarkers that predict the risk of UADT cancers.

## Supporting Information

Table S1 Impact of clinical factors on the association between the BRM promoter polymorphisms and upper aerodigestive tract cancers.

## References

[1] Wong KM, Hudson TJ, McPherson JD (2011). Unraveling the genetics of cancer: genome sequencing and beyond. Annu. Rev. Genomics Hum. Genet.

[2] Jones PA, Baylin SB (2007). The epigenomics of cancer. Cell.

[3] Reisman DN, Sciarrotta J, Bouldin TW, Weissman BE, Funkhouser WK (2005). The expression of the SWI/SNF ATPase subunits BRG1 and BRM in normal human tissues. Appl. Immunohistochem. Mol. Morphol.

[4] Simone C (2006). SWI/SNF: the crossroads where extracellular signaling pathways meet chromatin. J. Cell. Physiol.

[5] Klochendler-Yeivin A, Muchardt C, Yaniv M (2002). SWI/SNF chromatin remodeling and cancer. Curr. Opin. Genet. Dev.

[6] Morrison AJ, Shen X (2006). Chromatin modifications in DNA repair. Results Probl. Cell Differ.

[7] Peterson CL, Workman JL (2000). Promoter targeting and chromatin remodeling by the SWI/SNF complex. Curr. Opin. Genet. Dev.

[8] Wang W, Cote J, Xue Y, Zhou S, Khavari PA, Biggar SR (1996). Purification and biochemical heterogeneity of the mammalian SWI-SNF complex. EMBO J.

[9] Zhao Q, Wang QE, Ray A, Wani G, Han C, Milum K (2009). Modulation of nucleotide excision repair by mammalian SWI/SNF chromatin-remodeling complex. J. Biol. Chem.

[10] Gong F, Fahy D, Liu H, Wang W, Smerdon MJ (2008). Role of the mammalian SWI/SNF chromatin remodeling complex in the cellular response to UV damage. Cell Cycle.

[11] Zhang L, Chen H, Gong M, Gong F (2013). The chromatin remodeling protein BRG1 modulates BRCA1 response to UV irradiation by regulating ATR/ATM activation. Front. Oncol.

[12] Bennett G, Papamichos-Chronakis M, Peterson CL (2013). DNA repair choice defines a common pathway for recruitment of chromatin regulators. Nat. Commun.

[13] Wang W, Xue Y, Zhou S, Kuo A, Cairns BR, Crabtree GR (1996). Diversity and specialization of mammalian SWI/SNF complexes. Genes Dev.

[14] Bochar DA, Wang L, Beniya H, Kinev A, Xue Y, Lane WS (2000). BRCA1 is associated with a human SWI/SNF-related complex: linking chromatin remodeling to breast cancer. Cell.

[15] Liu G, Gramling S, Munoz D, Cheng D, Azad AK, Mirshams M (2011). Two novel BRM insertion promoter sequence variants are associated with loss of BRM expression and lung cancer risk. Oncogene.

[16] Mizutani T, Ito T, Nishina M, Yamamichi N, Watanabe A, Iba H (2002). Maintenance of integrated proviral gene expression requires *BRM*, a catalytic subunit of SWI/SNF complex. J. Biol. Chem.

[17] An HX, Claas A, Savelyeva L, Seitz S, Schlag P, Scherneck S (1999). Two regions of deletion in 9p23-24 in sporadic breast cancer. Cancer Res.

[18] Girard L, Zochbauer-Muller S, Virmani AK, Gazdar AF, Minna JD (2000). Genome-wide allelotyping of lung cancer identifies new regions of allelic loss, differences between small cell lung cancer and non-small cell lung cancer, and loci clustering. Cancer Res.

[19] Sarkar S, Roy BC, Hatano N, Aoyagi T, Gohji K, Kiyama R (2002). A novel ankyrin repeat-containing gene (kank) located at 9p24 is a growth suppressor of renal cell carcinoma. J. Biol. Chem.

[20] Tripathi A, Dasgupta S, Roy A, Sengupta A, Roy B, Roychowdhury S (2003). Sequential deletions in both arms of chromosome 9 are associated with the development of head and neck squamous cell carcinoma in indian patients. J. Exp. Clin. Cancer Res.

[21] Gunduz E, Gunduz M, Ali MA, Beder L, Tamamura R, Katase N (2009). Loss of heterozygosity at the 9p21-24 region and identification of BRM as a candidate tumor suppressor gene in head and neck squamous cell carcinoma. Cancer Invest.

[22] Reisman DN, Sciarrotta J, Wang W, Funkhouser WK, Weissman BE (2003). Loss of BRG1/BRM in human lung cancer cell lines and primary lung cancers: correlation with poor prognosis. Cancer Res.

[23] Glaros S, Cirrincione GM, Muchardt C, Kleer CG, Michael CW, Reisman D (2007). The reversible epigenetic silencing of BRM: implications for clinical targeted therapy. Oncogene.

[24] Yamamichi N, Inada K, Ichinose M, Yamamichi-Nishina M, Mizutani T, Watanabe H (2007). Frequent loss of *BRM* expression in gastric cancer correlates with histologic features and differentiation state. Cancer Res.

[25] Shen H, Powers N, Saini N, Comstock CE, Sharma A, Weaver K (2008). The SWI/SNF ATPase *BRM* is a gatekeeper of proliferative control in prostate cancer. Cancer Res.

[26] Decristofaro MF, Betz BL, Rorie CJ, Reisman DN, Wang W, Weissman BE (2001). Characterization of SWI/SNF protein expression in human breast cancer cell lines and other malignancies. J. Cell. Physiol.

[27] Strober BE, Dunaief JL, Guha S, Goff SP (1996). Functional interactions between the hBRM/hBRG1 transcriptional activators and the pRB family of proteins. Mol. Cell. Biol.

[28] Muchardt C, Bourachot B, Reyes JC, Yaniv M (1998). Ras transformation is associated with decreased expression of the BRM/SNF2alpha ATPase from the mammalian SWI-SNF complex. EMBO J.

[29] Fukuoka J, Fujii T, Shih JH, Dracheva T, Meerzaman D, Player A (2004). Chromatin remodeling factors and BRM/BRG1 expression as prognostic indicators in non-small cell lung cancer. Clin. Cancer Res.

[30] Yamamichi N, Yamamichi-Nishina M, Mizutani T, Watanabe H, Minoguchi S, Kobayashi N (2005). The *BRM* gene suppressed at the post-transcriptional level in various human cell lines is inducible by transient HDAC inhibitor treatment, which exhibits antioncogenic potential. Oncogene.

[31] Wang JR, Gramling SJ, Goldstein DP, Cheng D, Chen D, Azad AK (2013). Association of two BRM promoter polymorphisms with head and neck squamous cell carcinoma risk. Carcinogenesis.

[32] Jemal A, Siegel R, Ward E, Hao Y, Xu J, Thun MJ (2009). Cancer statistics, 2009. CA Cancer J. Clin.

[33] Zhou W, Liu G, Miller DP, Thurston SW, Xu LL, Wain JC (2002). Gene-environment interaction for the ERCC2 polymorphisms and cumulative cigarette smoking exposure in lung cancer. Cancer Res.

[34] Perez-Moreno P, Brambilla E, Thomas R, Soria JC (2012). Squamous cell carcinoma of the lung: molecular subtypes and therapeutic opportunities. Clin. Cancer Res.

[35] Cheng L, Alexander RE, Maclennan GT, Cummings OW, Montironi R, Lopez-Beltran A (2012). Molecular pathology of lung cancer: key to personalized medicine. Mod. Pathol.

[36] Gramling S, Rogers C, Liu G, Reisman D (2011). Pharmacologic reversal of epigenetic silencing of the anticancer protein BRM: a novel targeted treatment strategy. Oncogene.

