# Supplementary material for: Two BRM promoter insertion polymorphisms increase the risk of early-stage upper aerodigestive tract cancers
Source: Cancer Med. 2014 Feb 12;3(2):426–33. doi: 10.1002/cam4.201 (PMC3987092; doi:10.1002/cam4.201)
Supplement: Table S1 — Impact of clinical factors on the association between the BRM promoter polymorphisms and upper aerodigestive tract cancers. [file cam40003-0426-sd1.docx]

**SUPPLEMENTARY MATERIAL**

| **Supplementary Table 1. Impact of clinical factors on the association between the BRM promoter polymorphisms and upper aerodigestive tract cancers** | | |
| --- | --- | --- |
| **Clinical factor [1]** | **Adjusted OR (95% CI); n [2]** | |
|  | **Single homozygous BRM-741 or BRM-1321** | **Double homozygous BRM-741/-1321** |
| Age  Under 60  Over 60 | 1.45 (0.8-2.5); n=141  1.53 (0.6-2.8); n=194 | 2.53 (1.0-6.6); n=54  2.44 (1.0-6.3); n=66 |
| Sex  Male  Female | 1.42 (0.8-2.2); n=211  1.48 (0.6-2.8); n=124 | 2.39 (1.0-5.0); n=78  2.61 (0.9-8.3); n=32 |
| Smoking status  Current Smokers  Ex-smokers  Never-smokers | 1.47 (0.8-2.9); n=77  1.43 (0.8-2.8); n=142  1.39 (0.7-2.8); n=116 | 2.37 (0.7-8.1); n=29  2.44 (0.9-6.8); n=50  2.64 (1.0-7.4); n=41 |
| Histology [3]  Adenocarcinoma  Squamous cell carcinoma | 1.28 (0.8-2.1); n=262  1.52 (0.9-2.6); n=278 | 2.35 (1.0-4.5); n=89  2.64 (1.1-4.2); n=95 |
| Cancer type  Lung  Esophageal  Head and neck | 1.48 (0.9-2.9); n=155  1.17 (0.4-3.2); n=63  1.51 (0.8-3.0); n=117 | 2.58 (0.9-7.2); n=56  1.68 (0.6-8.8); n=23  2.71 (0.9-7.4); n=41 |
| Stage [4]  I  II | 1.42 (0.9-2.1); n=308  1.59 (0.7-4.2); n=237 | 2.54 (1.4-4.2); n=109  NA [3] |

[1] Subset analysis for family history was not performed due to the small number of individuals noting a positive family history.

[2] The OR was adjusted for: age, sex, smoking status, pack-years, and family history of UADT cancers.

[3] All controls were used for each analysis.

[4] There were too few Stage II cases to analyze the double homozygotes.
